# Untargeted plasma metabolomics identifies broad metabolic perturbations in glycogen storage disease type I

**DOI:** 10.1002/jimd.12451

**Published:** 2021-11-10

**Authors:** Tamara Mathis, Martin Poms, Harald Köfeler, Matthias Gautschi, Barbara Plecko, Matthias R. Baumgartner, Michel Hochuli

**Affiliations:** ^1^ Division of Endocrinology, Diabetes, and Clinical Nutrition University Hospital Zurich, University of Zurich Zurich Switzerland; ^2^ Department of Clinical Chemistry and Biochemistry University Children's Hospital Zurich, University of Zurich Zurich Switzerland; ^3^ Core Facility Mass Spectrometry Medical University of Graz Graz Austria; ^4^ Division of Pediatric Endocrinology, Diabetology and Metabolism, Department of Pediatrics and Institute of Clinical Chemistry University Hospital Bern, Inselspital Bern Switzerland; ^5^ Department of Pediatrics and Adolescent Medicine Medical University of Graz Graz Austria; ^6^ Division of Metabolism and Children's Research Center (CRC) University Children's Hospital, Zurich, University of Zurich Zurich Switzerland; ^7^ radiz—Rare Disease Initiative Zurich, Clinical Research Priority Program for Rare Diseases University of Zurich Zurich Switzerland; ^8^ Department of Diabetes, Endocrinology, Nutritional Medicine and Metabolism Inselspital, Bern University Hospital and University of Bern Bern Switzerland

**Keywords:** complications, glycogen storage disorder, GSD, lipids, metabolic disturbances, metabolomics

## Abstract

**Background:**

The metabolic defect in glycogen storage disease type I (GSDI) results in fasting hypoglycemia and typical secondary metabolic abnormalities (eg, hypertriglyceridemia, hyperlactatemia, hyperuricemia). The aim of this study was to assess further perturbations of the metabolic network in GSDI patients under ongoing treatment.

**Methods:**

In this prospective observational study, plasma samples of 14 adult patients (11 GSDIa, 3 GSDIb. Mean age 26.4 years, range 16‐46 years) on standard treatment were compared to a cohort of 31 healthy controls utilizing ultra‐high performance liquid chromatography (UHPLC) in combination with high resolution tandem mass spectrometry (HR‐MS/MS) and subsequent statistical multivariate analysis. In addition, plasma fatty acid profiling was performed by GC/EI‐MS.

**Results:**

The metabolomic profile showed alterations of metabolites in different areas of the metabolic network in both GSD subtypes, including pathways of fuel metabolism and energy generation, lipids and fatty acids, amino acid and methyl‐group metabolism, the urea cycle, and purine/pyrimidine metabolism. These alterations were present despite adequate dietary treatment, did not correlate with plasma triglycerides or lactate, both parameters typically used to assess the quality of metabolic control in clinical practice, and were not related to the presence or absence of complications (ie, nephropathy or liver adenomas).

**Conclusion:**

The metabolic defect of GSDI has profound effects on a variety of metabolic pathways in addition to the known typical abnormalities. These alterations are present despite optimized dietary treatment, which may contribute to the risk of developing long‐term complications, an inherent problem of GSDI which appears to be only partly modified by current therapy.

## Graphical abstract

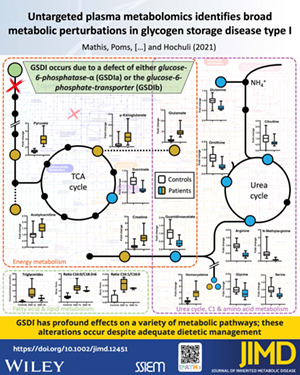

## 1 INTRODUCTION

Glycogen storage disease type I (GSDI, van Gierke's disease OMIM 232200) is an inherited metabolic disorder resulting from a defect of either glucose‐6‐phosphatase‐α (GSDIa) or the glucose‐6‐phosphate‐transporter (GSDIb), translocating glucose‐6‐phosphate into the lumen of the endoplasmic reticulum for hydrolysis by glucose‐6‐phosphatase. The primary metabolic abnormality of both GSDIa and Ib is fasting hypoglycemia, since glucose‐6‐phosphate produced via gluconeogenesis or glycogenolysis cannot be metabolized to glucose.^1^, ^2^

The mainstay of treatment in GSDI is a structured diet, consisting of a regular supply of (complex) carbohydrates to maintain normoglycemia (≥3.5‐4 mmol/L) and to control associated metabolic problems.^3^, ^4^ The enzyme defect leads to widespread metabolic disturbances. In either GSDIa or GSDIb, typical secondary biochemical abnormalities are hyperlactatemia/lactic acidosis, hypertriglyceridemia, hypercholesterolemia, and hyperuricemia, at least in part depending on the quality of dietary treatment (adherence to the diet) and glucose homeostasis, that is, the frequency of low‐blood glucose or hypoglycemia, respectively. Hepatic cytoplasmic accumulation of glucose‐6‐phosphate and subsequent increases of other phosphomonoesters (ie, glycolytic intermediates) trigger a series of changes in metabolic fluxes, such as enhanced glycolytic flux with production of excessive lactate, and markedly increased hepatic de novo lipogenesis (from increased availability of substrates, but also by increased expression and activity of lipogenic transcription factors) with profound changes in lipid metabolism and liver steatosis.^5^, ^6^, ^7^, ^8^ ^,^ ^10^, ^11^, ^12^, ^13^, ^14^

These secondary metabolic changes occur despite a physiological downregulation of insulin secretion in response to hypoglycemia, as a hormonal regulator of glycolytic flux and lipid synthesis.^10^, ^14^ Accumulation of phosphorylated (glycolytic) intermediates has been shown to result in depletion of hepatic Pi and ATP^11^ and may cause a disturbed cellular energy state. Moreover, recent findings suggest impaired or limited mitochondrial capacity in animal models of GSDI, with, for example, impaired oxidative phosphorylation and reduced numbers of functional mitochondria.^9^, ^16^ Overall, these changes in fuel and energy metabolism may have subsequent effects on a variety of metabolic processes. With time, typical long‐term complications of GSDI such as liver adenomas or nephropathy often develop, even in patients with apparently stable metabolic control. Although the quality of metabolic (glucose) control may be a modifying factor for the development and progression of these complications, they appear to be inherent to the metabolic disorder. However, the molecular mechanisms and specific risk factors underlying these complications are still incompletely understood. Studies in animal models showed that lipid accumulation in liver and kidney with disturbed autophagy may be contributing factors.^17^, ^18^, ^19^ Currently, there remains a gap of knowledge regarding specific elements of therapy that are most important to avoid or delay the progression of complications in order to achieve a good long‐term outcome, or biomarkers that will guide us to provide optimal treatment. Further advances in understanding the underlying metabolic perturbations in GSDI are necessary to support these efforts to deliver optimal care. The goal of the present study was to delineate areas of disturbed metabolic function in both GSDI subtypes under the established ongoing treatment by using plasma metabolomics, and to evaluate whether laboratory parameters that are routinely used in the clinical follow‐up and monitoring of patients (such as plasma triglycerides and lactate) would relate to these disturbances identified in the plasma metabolome. Furthermore, we assessed whether the metabolic alterations would relate to the presence or absence of the typical long‐term complications (liver adenomas or nephropathy).

## 2 MATERIALS AND METHODS

### 2.1 Study design

The study was designed as a prospective observational study, and was performed under the ongoing established routine care of the patients. No study‐specific treatment or therapeutic intervention were performed. Blood samples were collected as part of routine medical care during a planned period of 2 years. Lithium‐heparin plasma samples for untargeted metabolomics and plasma fatty acid profiles were obtained at regular outpatient consultations during the study period, along with blood work for standard laboratory monitoring for GSDI. Venous blood samples were collected 2 to 4 hours after the last meal/snack (mainly in the late morning before lunch). In healthy controls, a single blood sample 3 to 5 hours after the last meal (mainly in the late morning prior to lunch) was collected for measurement of plasma metabolome and fatty acid profiles, as well as routine parameters of clinical chemistry. All procedures were in accordance with the ethical standards of the responsible committee on human experimentation (registration number KEK ZH 2013‐0632, PB_2016‐01114) and with the Helsinki Declaration of 1975 as revised in 2000.

### 2.2 Patients

Male or female patients >16 years with GSDIa or GSDIb were eligible for this study. Fourteen patients (11 GSDIa, 3 GSDIb) were recruited from the adult metabolic clinics of the University Hospitals in Zurich, Bern and Basel, and the Cantonal Hospital St. Gallen (Switzerland), on the occasion of a regular consultation. Thirty‐one age‐matched healthy controls were recruited at the University Hospital Zurich, via advertisements posted around the university campus. Informed consent was obtained from all patients and healthy controls included in the study. The first patient was included in November 2014; the last patient completed the study in April 2017.

### 2.3 Laboratory measurements

Non study specific parameters of routine clinical chemistry were measured at the accredited clinical chemistry laboratory of the local hospital. Blood samples were centrifuged at 3000 rpm for 10 minutes immediately after blood collection, and separated plasma was stored frozen at −80°C until analysis. Shipment was on dry ice and plasma metabolomics was performed in one center (University Children's Hospital Zurich).

### 2.4 Fatty acid profiles

Fatty acid profiles (GC‐EI/MS of total fatty acids, free plus esterified): 50 μL of human plasma were diluted with 5 mL of methyl‐tert‐butylether (MTBE) and 1.5 mL of methanol. Each sample was spiked with 400 nmol FA 17:0 as internal standard immediately. Then, lipids were extracted according to Matyash et al.^20^ Lipid extracts were dried and dissolved in 1 mL methanolic NaOH. After 10 minutes incubation at 80°C, samples were cooled for 5 minutes on ice. Then, 1 mL boron trifluoride (BF_3_) was added and samples were incubated for 10 minutes at 80°C. Fatty acid methyl esters were extracted with 1 mL saturated NaCl and 2 mL hexane. The hexane phase was dried and methyl esters dissolved in 1.5 mL hexane. An Agilent GC‐MSD 5977 equipped with a TR‐FAME 30m column was used for analysis. Helium was used as carrier gas at a flow of 1 mL/min, in split mode, at 250°C injector temperature. The initial oven temperature of 150°C was held for 0.5 minute and then the temperature was increased to 180°C at a rate of 10°C/min. This was followed by a further increase to 190°C at a rate of 0.5°C/min and then increased to 250°C at a rate of 40°C/min and kept for 3 minutes. The mass spectrometer was run in electron impact mode and fatty acids were detected in full scan of m/z 80 to 400. Source temperature was set to 300°C and the transfer line temperature to 200°C. Peak areas for FAs were calculated by MassHunter and related to FA 17:0 internal standard peak areas. Quantities for FAs were calculated by a one point calibration of the individual FAs vs the known amount of FA 17:0 (internal standard) including an individual response factor for each targeted FA.

### 2.5 Untargeted plasma metabolomics

Samples for untargeted metabolomics were prepared by mixing 100 μL plasma with 400 μL ice‐cold methanol, vortexing and subsequent centrifugation (14'500 rpm, 15 minutes, 4°C). Four hundred and fifty microliters of the supernatant was evaporated on a Concentrator plus (Eppendorf, Hamburg, Germany) for 1 hour until dry and redissolved in 200 μL 50% MeOH, including 0.1 mM uracil‐5‐d_1_ as an internal standard, which was used a qualitative control. Chromatographic separation was achieved using a 2.1 × 100 mm Kinetex HILIC column (Phenomenex, Torrance, California) with a stepwise gradient from 100% buffer B (acetonitrile/water 95:5, 0.1% formic acid, 5 mM ammonium formate) to 100% buffer A (acetonitrile/water 50:50, 0.1% formic acid, 5 mM ammonium formate), 0% to 50% A over 12 minutes, 50% to 100% A over 3 minutes with a total runtime of 20 minutes. Flow rate was kept constant at 0.4 mL/min with a column temperature of 30°C. Samples were measured in randomized order in single batches for positive and negative ionization mode, respectively, with a blank injection every six runs.

Mass spectra were acquired using a heated electro‐spray ionization (HESI) source of a Q‐Exactive high resolution, accurate mass spectrometer (Thermo Scientific, Waltham, Massachusetts). Mass spectra were recorded in positive and negative mode with the MS detector in full‐scan mode (Full‐MS) in the scan‐range 67 to 1000 *m/z* with data‐dependent (dd‐MS2) acquisition of fragment ions from the top‐5 most abundant ions per scan. Detailed MS parameters can be found in Appendix S1.

Raw data were assembled using Xcalibur (4.1, Thermo) and converted to .mzXML format using the MSconvert.exe program.^21^ Data preprocessing, including baseline correction and peak alignment as well as peak picking was achieved utilizing the XCMS package^22^ in R(R Core Team. R: A Language and Environment for Statistical Computing. R Foundation for Statistical Computing Vienna, Austria [2016].) (x64, v3.3.1). Detailed xcms parameters can be found in Appendix S1.

Data pretreatment included noise filtering and missing data imputation, where features missing from at least 75% of measurements were excluded, and total ion current normalization, where the sum of all features is compared to the average of all runs and converted to a scaling factor for each feature, additionally to pareto scaling. Each feature is then converted to a fraction of the sum total. Data pretreatment was carried out in R using the muma software package (version 1.4).^23^ Detailed muma parameters can be found in Appendix S1.

### 2.6 Statistical analysis

Multivariate analyses were carried out in SIMCA v13.0.3 (Unimetrics, Malmö, Sweden). Unsupervised principal component analysis (PCA) was applied to identify significantly altered features. The comparison GSDI patients vs age‐matched controls was carried out for positive as well as negative mode. Multivariate analysis for the comparisons of GSDIa vs GSDIb patients, patients with liver adenoma vs patients without liver adenoma, and patients with microalbuminuria vs patients without microalbuminuria did not yield significant results and were therefore evaluated using univariate analysis.

Features that exhibited the largest changes were mined utilizing a pathway‐ and network‐based approach using the mummichog algorithm.^24^ Tentative feature annotation from mummichog was subsequently confirmed by comparison to an internal database consisting of roughly 400 metabolites, or retention time analysis and fragment pattern matching against the Metlin,^25^ HMDB,^26^ and mzCloud databases for metabolites that were not in the library.

Fold changes were calculated as the ratio of the mean peak area of the respective feature and *P* values were calculated by independent two‐tailed *t* tests on the mean peak area for any given metabolite using univariate analysis. Due to multiple hypothesis testing, *P* values were adjusted using Bonferroni correction to evaluate significance. Furthermore, correlations between metabolites were calculated by determining the Spearman correlation coefficient within as well as across different experiments (laboratory measurements, metabolomics, and fatty acid profiles) for all subjects as well as all subgroups (controls, patients, GSDIa, GSDIb), respectively.

## 3 RESULTS

Fourteen patients with glycogen storage disease type I (GSDI, 11 GSDIa, 3 GSDIb) were compared to a cohort of 31 age‐matched healthy controls (Table 1). All patients had typical, enzymatically, or/and genetically confirmed GSDI, and followed a standard treatment with regular carbohydrate intake. Most patients restricted galactose and fructose. Twelve patients (10 GSDIa, 2 GSDIb) used uncooked corn starch to maintain normoglycemia during nighttime, and two patients (1 GSDIa, 1 GSDIb) received continuous nocturnal gastric tube feeding with glucose polymer.

**TABLE 1:** Clinical description of the patient and control cohorts

	Patients	GSD Ia	GSD Ib	controls
Total	14	11	3	31
Gender (m, f)	11 m, 3 f	9 m, 2 f	2 m, 1 f	16 m, 15 f
Age (years)	26.9 ± 9.6	27.0 ± 10.2	26.7 ± 8.9	30.1 ± 9.3
Weight (kg)	67.0 ± 13.1	68.2 ± 12.2	62.9 ± 18.5	69.2 ± 10.0
Height (cm)	166.2 ± 9.3^a^	168.4 ± 7.6	158.3 ± 12.7	173.9 ± 8.2
BMI (kg/m^2^)	24.2 ± 4.3	24.0 ± 3.4	25.1 ± 7.9	22.7 ± 2.1
Triglycerides (mmol/L)	7.6 ± 5.1^a^	8.8 ± 5.2	3.5 ± 2.4	0.8 ± 0.4
Total cholesterol (mmol/L)	6.4 ± 2.7^a^	7.3 ± 2.3	3.3 ± 0.4#	4.4 ± 1.0
AST (U/L)	72.0 ± 51.8^a^	79.3 ± 53.7	47.5 ± 44.0	24.1 ± 4.7
ALT (U/L)	73.0 ± 44.8^a^	79.1 ± 43.7	52.8 ± 51.5	19.5 ± 7.8
gGT (U/L)	143.0 ± 84.8^a^	162.1 ± 88.0	79.4 ± 19.7#	18.5 ± 12.9
AP (U/L)	95.1 ± 41.5^a^	85.0 ± 38.6	128.6 ± 37.9#	54.7 ± 18.1
Biotinidase (nmol/min/mL)	12.6 ± 2.0^a^	12.6 ± 1.9	12.8 ± 2.6	7.8 ± 1.6
Lactate (mmol/L)	5.3 ± 2.2	5.2 ± 2.5	3.8 ± 2.7	n.a.
Serum glucose (mmol/L)	5.4 ± 0.8^a^	5.5 ± 0.7	4.8 ± 1.2	4.6 ± 0.3
Serum creatinine (μmol/L)	56.7 ± 15.1^a^	57.9 ± 13.7	52.9 ± 22.3	75.3 ± 10.3
Microalbuminuria	8/14 patients	6/11 patients	2/3 patients	
Liver adenomas	9/14 patients	7/11 patients	2/3 patients	

^a^ Significantly different GSDI vs healthy controls. #significantly different GSDIb vs Ia.

**FIGURE 1:**
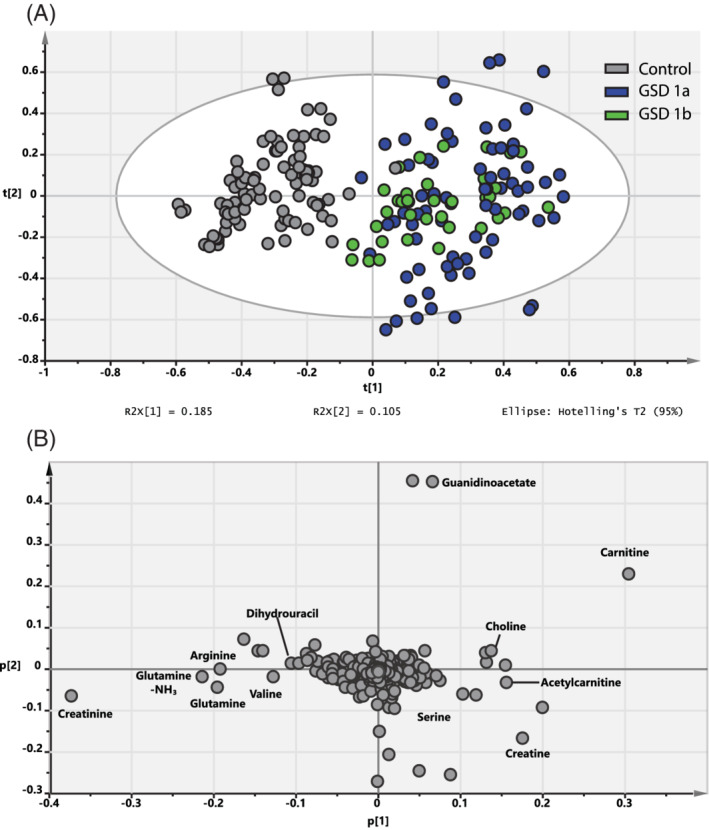
(A) Unsupervised principal component analysis (PCA) of GSDIa (blue) and GSDIb (green) patient plasma samples vs healthy controls (gray points) in positive mode with R2X[1] displaying the interpretable degree of the first principle component (horizontal) and R2X[2] displaying the interpretable degree of the second principle component (vertical). All samples are shown as technical triplicates. (B) PCA loading plot. Selected components, which could be confirmed by comparison to the internal library or fragment pattern matching are annotated

Metabolomics analysis of GSD patient plasma samples vs healthy controls yielded 1687 and 3207 features in total for positive and negative mode, respectively, with 235 and 1571 statistically significant features after Bonferroni correction. Principal component analysis (PCA) displayed metabolomic variation and good segregation between the two groups (Figure 1) with 83% of the variation within the training set explained by the model and 34% of the variation in the training set predicted by the model according to cross validation in the 22nd component (R2X (cum) = 0.83; Q2 (cum) = 0.34) in positive mode. The PCA plot for negative mode can be found in Figure S1 (22nd component; R2X (cum) = 0.73; Q2 (cum) = 0.58). Data analysis showed no inherent bias with regard to gender, medical center where samples were collected or the type of overnight nutrition regimen (cornstarch vs continuous nocturnal gastric tube feeding).

**FIGURE 2:**
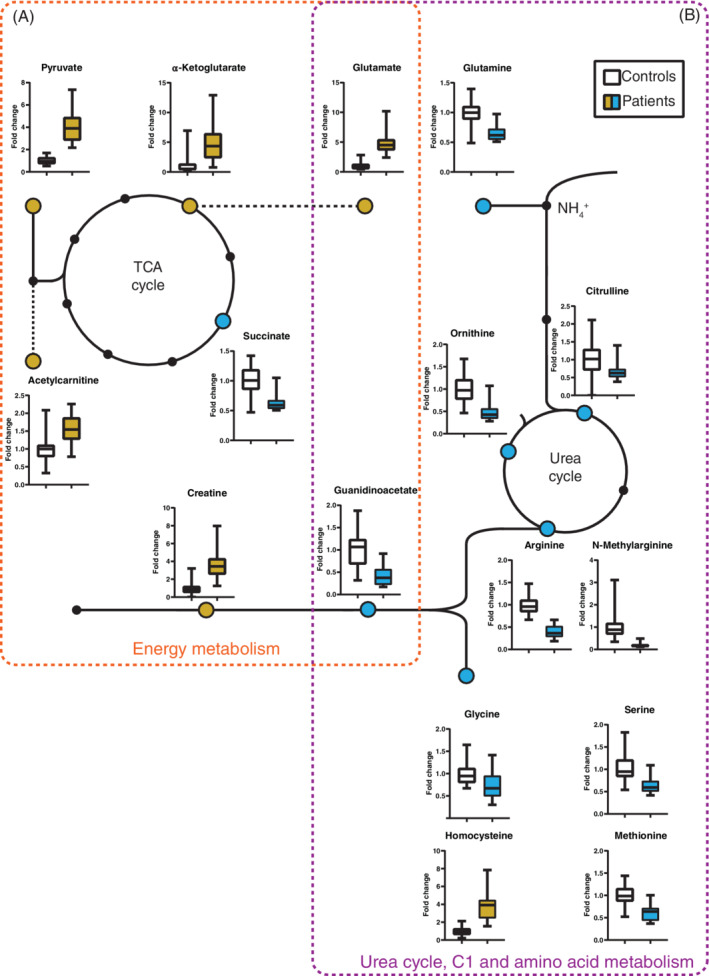
Significantly altered metabolites associated with energy metabolism (A) as well as amino acid, C1 and urea cycle metabolism (B). Metabolites that are increased in GSDI patients compared to controls are shown in yellow, decreases in blue

The metabolic profile of GSD patients showed numerous alterations of metabolites in different areas of the metabolic network, such as glycolysis and the tricarboxylic acid cycle, in lipid and fatty acid metabolism, in the metabolism of creatine, in the urea cycle, in the amino acid and methyl (C1) group metabolism, purine/pyrimidines, but also changes of cofactors such as biotin (Figure 2 and Tables 2 and S1).

**TABLE 2:** Excerpt of significantly altered metabolites in GSDI patients compared to controls. The full table including fragments and adducts can be found in Table S1

Feature	Ion	Metabolite	Fold change	Corr. *P* value
*Energy metabolism*				
204.12292	M + H	Acetylcarnitine	1.47	7.98E‐08
145.01257	M − H	α‐Ketoglutarate	4.44	8.23E‐10
132.0748	M + H	Creatine	3.73	5.93E‐24
114.06205	M + H	Creatinine	0.63	2.35E‐17
87.00701	M − H	Pyruvate	3.7	3.17E‐34
117.01756	M − H	Succinate	0.66	5.99E‐16
148.06057	M + H	Glutamate^a^	4.67	3.34E‐27
162.10734	M + H	Carnitine	1.24	3.48E‐04
*Urea cycle, C1 and amino acid metabolism*				
175.11881	M + H	Arginine	0.42	6.51E‐33
176.10215	M + H	Citrulline	0.68	4.56E‐03
133.09458	M + H	Ornithine	0.5	3.87E‐17
147.07446	M + H	Glutamine	0.72	1.75E‐09
189.12762	M + H	N‐Methylarginine^b^	0.22	3.56E‐20
118.05844	M + H	Guanidinoacetate^a^	0.43	2.36E‐21
106.04542	M + H	Serine	0.62	6.03E‐16
150.05564	M + H	Methionine	0.63	1.22E‐16
136.04212	M + H	Homocysteine	3.94	7.87E‐26
76.03368	M + H	Glycine	0.78	5.93E‐03
*Purines and pyrimidines*				
268.09561	M + H	Adenosine	0.3	9.73E‐03
244.08633	M + H	Cytidine	3.19	2.06E‐19
115.04332	M + H	Dihydrouracil	0.73	4.41E‐03
249.0859	M + H	Thymidine	0.36	2.67E‐05
127.04128	M + H	Thymine	0.81	2.40E‐02
139.04438	M + H	Urocanate	0.68	8.90E‐03
151.0209	M − H	Xanthine	7.52	5.47E‐13
*Others*				
245.08444	M + H	Biotine	4.35	6.03E‐19

^a^ Several metabolites occupy multiple metabolic functions and therefore cannot be uniquely allocated to one specific category.

^b^ Metabolites that were not in the internal library and were confirmed by fragment pattern matching.

**FIGURE 3:**
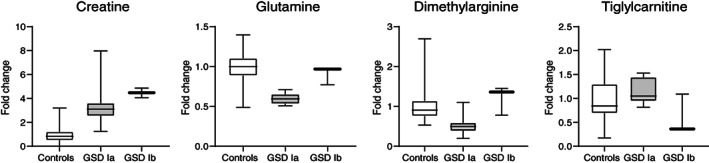
Metabolites that exhibit significant differences between patients with GSDIa vs GSDIb

Most alterations were seen consistently in patients of both GSD subtypes (Ia and Ib), but a few metabolites segregated between the two subtypes, such as creatine or glutamine (Table S2 and Figure 3). In general, there were no relevant differences of the analyzed metabolites in GSDI patients with or without liver adenomas, or microalbuminuria (Tables S3 and S4). Alterations of metabolites identified by untargeted metabolomics were not correlated to the concentrations of plasma glucose, triglycerides or lactate measured at the time of blood sampling (with the exception of the correlation of lactate with alanine), that is, parameters used to assess metabolic control in daily clinical practice. Average plasma glucose was normal (Table 1).

**FIGURE 4:**
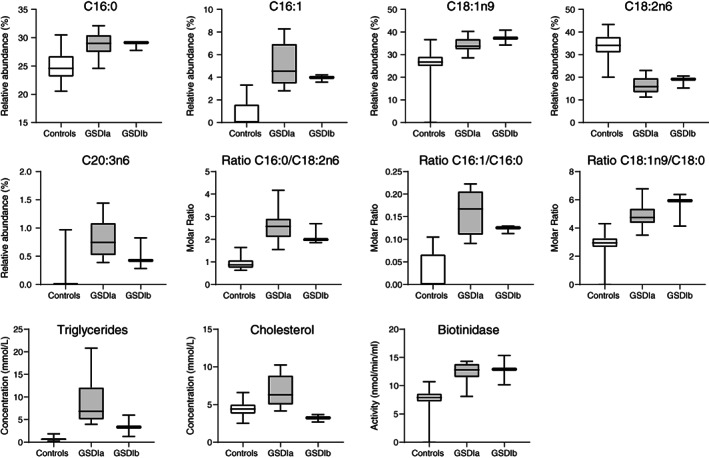
Fatty acid profiles from patients with GSDIa and GSDIb compared to controls. Relative abundance expresses the relative contribution of an individual fatty acid to the total fatty acid pool, while molar ratios express the ratio of two individual fatty acids. Panels in the bottom row show plasma triglycerides, total cholesterol and biotinidase activity

Regarding metabolism of fatty acids and lipids, GSDI patients show the typical abnormalities of the traditional lipid profile with hypertriglyceridemia and hypercholesterolemia (Table 1). In the plasma fatty acid profile, the relative abundance of palmitate (16:0) was increased, whereas the proportion of linoleic acid (18:2) was decreased. The molar ratio palmitate/linoleate (16:0/18:2) can serve as marker for de novo lipogenesis.^27^ This marker was clearly increased across this cohort, to a similar extent in both GSDI subtypes. Along with this finding, an increased proportion of monounsaturated fatty acids synthesized via d9‐desaturase (eg, palmitoleic acid 16:1, and oleic acid 18:1, including ratios 16:1/16:0 and 18:1/18:0) was observed, a marker that is also associated with de novo lipogenesis (Figure 4 and Table S5). These markers of lipogenesis (16:0/18:2, 16:1/16:0, 18:1/18:0) did not correlate with plasma triglyceride levels, nor the plasma glucose and lactate concentrations. Furthermore, the presence of liver adenomas was not associated with specific features/alterations in the fatty acid profile, such as higher levels of lipogenesis markers. However, there was an increased proportion of palmitoleic acid, that is, the ratio 16:1/16:0 (0.185 vs 0.120, *P* = 0.031) and a trend for higher total cholesterol levels (8.72 vs 6.36 mmol/L, *P* = 0.097) in the subgroup of GSDIa patients with microalbuminuria. The proportion of di‐homo‐γ‐linoleic acid (20:3n6) as a downstream metabolite of α‐linoleic acid was increased, suggestive of increased d6‐deasaturase activity. Alongside with these alterations in the fatty acid profile, biotin as a cofactor of various carboxylases, for example involved in de novo lipogenesis or gluconeogenesis, was increased in GSDI, and biotinidase activity as an enzyme involved in the recycling of biotin was elevated (Figure 4). Metabolites associated with biotin availability were not significantly different from controls. Biotinidase activity or biotin did not correlate with markers of de novo lipogenesis in the fatty acid profile (eg, 16:0/18:2).

## 4 DISCUSSION

The present study demonstrates that the metabolic defect of GSDI results in broader disturbances of the metabolic network than previously known, notably in the areas of fuel and energy metabolism, lipids and fatty acids, amino acid and methyl(C1)‐group metabolism, intermediates of the urea cycle, and purine/pyrimidines. The observed alterations were present despite adequate dietary treatment, and did not correlate with plasma triglycerides or lactate, both parameters routinely used in clinical practice to assess metabolic control, nor with the actual venous plasma glucose concentration. Furthermore, most alterations detected by metabolomic analysis were present irrespective of the presence or absence of typical complications such as liver adenomas or nephropathy (micro‐albuminuria). With few exceptions, most of the observed metabolic alterations occurred in both GSD subtypes, although some to a different extent. A limited set of metabolites clearly segregated between the two subtypes.

Many of the observed metabolic alterations in GSDI are secondary to widespread changes in fuel and energy metabolism. Our results indicate that impaired or limited mitochondrial capacity identified in GSDI animal models may also be an element of the pathophysiology of the disease in humans. The combination of enhanced fluxes through glycolysis combined with impaired or limited mitochondrial capacity may result in an altered energy state of the cell with profound effects on a variety of metabolic reactions. The observed metabolomic profile in our cohort is consistent with this hypothesis. Increased levels of creatine suggest an alteration of intracellular energy state, and confirm previous findings obtained in GSDIa patients by NMR spectroscopy.^28^ Elevated plasma levels of creatine have been found as a marker in a number of disorders with impaired mitochondrial function.^29^ Increased levels of pyruvate, acetylcarnitine, lactate, and α‐ketoglutarate would be in line with an enhanced glycolytic flux and some limitation or overflow (mismatch) of the TCA flux, respectively. Increased levels of α‐ketoglutarate, paralleled by decreased levels of succinate further downstream the pathway, may suggest some limitation of TCA flux between these intermediates (at the level of α‐ketoglutarate dehydrogenase). α‐Ketoglutaric aciduria has previously been observed in GSDI patients.^30^, ^31^ Glutamate was increased concomitant with α‐ketoglutarate in our cohort, which possibly results from direct interconversion, whereas glutamine levels were decreased in GSDIa. As a hypothesis, this observation may be due to increased glutaminolysis by the action of glucagon to stimulate mitochondrial anaplerotic flux in the setting of hypoglycemia, feeding the pool of glutamate (and α‐ketoglutarate).^32^ Alternatively, glutamine reductive carboxylation has been proposed as an alternative pathway for glutamine catabolism and as a mechanism for cytosol confined NADH recycling in case of mitochondrial dysfunction.^33^ It is not known whether this pathway would be active in GSDI as a means to regenerate cytosolic NADH to support the enhanced glycolytic flux and to ensure sufficient ATP yields from glycolysis. Altered intrahepatic phosphate levels due to the accumulation of phosphate esters and changes of the intracellular energy state have been described as triggering factors resulting in disturbed purine metabolism with increased uric acid production and hyperuricemia.^11^, ^34^, ^35^ Possible mechanisms underlying the observed alterations in pyrimidine metabolites remain to be elucidated. Increased cytidine levels have been associated with disorders of mitochondrial activity.^29^

Profound changes in lipid metabolism with hepatic steatosis are a hallmark of the disease.^5^, ^6^, ^7^, ^12^ In the present cohort, the plasma fatty acid profile of GSDI very much resembles the metabolic signature of patients with NAFLD/NASH, with increased markers of de novo lipogenesis and d9‐desaturase activity for example.^27^, ^36^ Increased production of (mono‐)unsaturated fatty acids via d9‐desaturase is commonly observed in conditions with enhanced lipid synthesis. Notably, the markers of hepatic de novo lipogenesis were not correlated to the degree of hypertriglyceridemia, and enhanced lipogenesis was observed both in patients with a milder or more severe phenotype. Furthermore, markers of de novo lipogenesis were similarly increased in both GSD subtypes, despite lower plasma triglyceride and cholesterol levels in GSDIb. These observations are inline with the concept that the hepatic VLDL production rate (ie, production of plasma triglycerides) in GSDI is not directly linked to the uniformly enhanced rate of de novo lipogenesis and the development of liver steatosis.^5^, ^37^ Although these features are present in both GSDI subtypes, GSDIb displays specific differences of lipid metabolism, for example, regarding atypical deoxysphingolipids.^12^, ^31^, ^38^ Dysregulation of hepatic lipid metabolism with impaired autophagy in liver cells has been proposed as a possible mechanism favoring adenoma formation in GSDI mouse models.^17^, ^39^ Moreover, overfeeding of GSDI mice (liver G6pc−/−) with a diet containing high amounts of the lipogenic substrates fructose or sucrose induced more rapid development of liver adenomas.^40^ Dysregulation of lipid metabolism may also play a role in the development of nephropathy.^18^, ^19^ With time, a majority of adult GSDI patients develop liver adenomas or some degree of nephropathy, although the extent and time of onset span a wide range. Persistently enhanced de novo lipogenesis may be one of the risk factors for the development of these complications, which is only partly modified by dietary treatment. Enhanced lipogenesis with increased activity of enzymatic reactions requiring biotin may also be an element underlying the increased biotinidase activity in plasma, which is a typical finding in most GSDI patients.^41^ Increased biotin levels in our cohort may be a direct consequence of enhanced recycling of biotin by biotinidase.

Disturbed patterns of amino acids or metabolites involved in the metabolism of methyl groups may also be associated with the presence of hepatic steatosis. Similar features have been observed in mouse NAFLD disease models.^42^ GSDI patients displayed a metabolite pattern of increased homocysteine levels along with decreased methionine and serine, which may suggest impaired remethylation processes, very similar to the metabolite constellation observed in the NAFLD mouse model. Altered serine availability also appears to be a determinant for the synthesis of atypical, potentially neurotoxic deoxysphingolipids, a pathomechanism that appears to be present also in GSDI.^12^ As a possible consequence of disturbances in the metabolism or transfer of methyl groups, the levels of the methylated arginine derivative N‐monomethylarginine (L‐NMMA) were clearly decreased in GSDI. Endogenous L‐NMMA essentially derives from methylated arginine residues in proteins, and protein methylation by protein arginine methyltransferases (PRMTs) may be altered in chronic (hepatic) disease or hepatic steatosis.^42^ L‐NMMA is an inhibitor of NO‐synthase (ie, of NO‐mediated vasodilation), and increased levels have been associated with the presence of vascular disease.^43^, ^44^ Whether decreased L‐NMMA levels as observed here in GSDI would confer a benefit regarding the development of (cardio‐)vascular disease (eg, in the setting of the marked dyslipidemia in GSDI) is not known. Disturbances in the remethylation pathway have also been shown to decrease glutathione as an important redox‐buffer in liver extracts of NAFLD mice.^42^ In contrast, hepatic glutathione levels were increased in a GSDI mouse model^8^, although the experimental setups cannot be directly compared. Glutathione could not be reliably detected in plasma by our experimental approach. The role of glutathione in the pathophysiology of GSDI, especially with regard to hepatic tumorgenesis is not clearly defined yet. Interestingly, decreased glutathione levels have been detected in human GSDI fibroblasts, although these cells do not represent the organs primarily affected by the metabolic defect and do not express glucose‐6‐phosphatase‐α.^45^

The urea cycle intermediates citrulline, arginine, and ornithine were decreased, suggesting reduced urea cycle flux/load. Although it has been shown that the activity of carbamoyl‐phosphate synthetase 1 (CPS1), the enzyme catalyzing the entry reaction of nitrogen to the urea cycle, may be downregulated in hepatic steatosis^46^ and therefore possibly also in GSDI, reduced urea cycle load may result from lower protein intake as a consequence of the specific diet primarily focusing on frequent and regular carbohydrate intake, which may also limit endogenous protein degradation in the frequent anabolic postprandial periods. However, the patterns of macronutrient intake in other GSDI cohorts were not characterized by a lower protein intake compared to age and gender matched peers.^47^ Ammonia levels are typically normal in GSDI (unpublished results).

Although good dietary treatment is the cornerstone of therapy to achieve good glycemic control and to improve or limit the typical secondary metabolic abnormalities such as hypertriglyceridemia, hyperlactatemia, or hyperuricemia, the treatment will not completely restore these metabolic alterations. The present study is performed in a real‐world setting under the established ongoing treatment, and confirms that numerous metabolic perturbations persist in different areas of the metabolic network despite adequate therapy, across a cohort with a broad spectrum of disease manifestation and fasting tolerance. Notably, the average plasma glucose across the cohort was normal at the time of sample collection. Although good metabolic (glycemic) control may modify the risk of progression or development of typical long‐term complications such as liver adenomas or nephropathy (albuminuria), they frequently do develop in patients with apparently good metabolic control. In a cross‐sectional analysis, the presence of liver adenomas or microalbuminuria was associated with more frequent episodes of low blood glucose in continuous glucose measurements (CGM).^48^ In other cohorts, the degree of hypertriglycridemia was associated with the progression of liver adenomas, or the presence of microalbuminuria.^49^, ^50^ In the present analysis, most detected metabolomic alterations were present irrespective of the presence or absence of these complications. Metabolic disturbances which are inherent to the metabolic disorder and that are only partly modified by treatment may at least in part confer a persistent risk for the development of complications.

The present study has several limitations. (a) There are limitations due to the number of study participants, for example, for assessing associations of the metabolic phenotype with the presence of complications. In addition, the limited number of GSDIb patients participating in this study restricts the statistical power to assess differences in the metabolic phenotype between the two subtypes. (b) It cannot be excluded that some observations in the metabolome may be related to the diet rather than the specific metabolic defect. However, with the exception of a regular carbohydrate intake and some limitation of fructose and galactose intake, patients do not follow a structured diet and there is broad interindividual variation of dietary habits, in contrast to the clear and homogenous segregation of the described features of the metabolome in GSDI compared to healthy controls. Furthermore, the combination of metabolic findings observed in GSDI cannot be explained by a specific pattern of nutrient intake inherent to the treatment of this metabolic defect.^47^ (c) Alterations of plasma metabolites will not directly reflect intracellular processes. However, many of the findings can be explained within the framework of (biochemical) mechanisms observed in animal models as mentioned previously, or targeted interventions in humans using isotope labeled tracers or liver 31P‐MR spectroscopy. (d) Findings from the untargeted metabolomics data were not complemented by targeted metabolite analysis. A targeted approach based on the present results will be chosen for our future studies. (e) The selection of sample preparation protocols as well as chromatography and mass spectrometry parameters in a metabolomics experiment act as a limiting factor in terms of which classes of metabolites can be observed, based on their chemical characteristics. The focus of this study on small polar metabolites, complemented by quantitative fatty acid characterization, therefore cannot rule out metabolic disturbances in other parts of the metabolome.

In summary, the metabolic defect of GSDI has profound effects on a variety of metabolic pathways in both GSDI subtypes, in addition to the known typical secondary metabolic abnormalities. Plasma triglycerides and lactate as commonly used markers to monitor metabolic control in clinical practice will only partly capture the manyfold metabolic disturbances. The hypotheses generated by the present exploratory study should direct future mechanistic studies in appropriate model systems. Performing regular blood glucose measurements to achieve stable glycemic control remains a primary element to guide the treatment, as the disturbance of glucose homeostasis by the defective action of glucose‐6‐phosphatase stands at the outset of a cascade of metabolic alterations. Combining (targeted) metabolome analysis with CGM in larger cohorts will give further insights into the mechanics of the metabolic network, and may reveal elements of metabolic disturbance that are particularly sensitive to glycemic control, the quality of dietary treatment, or the effect of pharmacological interventions, with the ultimate goal to identify suitable biomarkers that may guide us to provide optimized care.

## CONFLICT OF INTEREST

Tamara Mathis, Martin Poms, Harald Köfeler, Matthias Gautschi, Barbara Plecko, Matthias R. Baumgartner, and Michel Hochuli declare that they have no conflict of interest.

## INFORMED CONSENT

Informed consent was obtained from all patients and healthy controls included in the study.

## Supporting information


**Appendix S1**: Supporting Information
