# Supplementary material for: Untargeted plasma metabolomics identifies broad metabolic perturbations in glycogen storage disease type I
Source: J Inherit Metab Dis. 2021 Nov 10;45(2):235–47. doi: 10.1002/jimd.12451 (PMC9299190; doi:10.1002/jimd.12451)
Supplement: Supplementary file 1 — Appendix S1: Supporting Information [file JIMD-45-235-s001.docx]

**Supplementary Material**

*MS parameters and analysis settings:*

HESI parameters: sheath gas flow rate 35 arbitrary units (AU), aux gas flow rate 35 AU, sweep gas flow rate 2 AU, spray voltage 3.5 kV, capillary temperature 350°C, aux gas heater temperature 350°C. Detector settings for full MS: In-source CID 0.0 eV, µscans = 1, resolution = 70,000, AGC target 1e^6^, max IT = 35 ms, spectrum data type, profile. Detector setting for dd-MS2 were: µscans = 1, resolution = 17,500, AGC target 1e^5^, max IT = 80 ms, loop count = 5, isolation window 4.0 *m/z*, NCE 30.0, intensity threshold 1.3e^4^, apex trigger 2 to 4s, spectrum data type, profile.

XCMS parameters: xcmsSet (method=”centWave”, peakwidth= c(10, 120), ppm=2, noise=0, snthresh=10, mzdiff=0.001, prefilter=c(3, 100), mzCenterFun=”xMean”, integrate=2, fitgauss=F); retcor(method=”obiwarp”, plottype=”deviation”, distFunc=”cor”, profStep=1, response=2, gapInit=0.6, gapExtend=2.7, factorDiag=2, FactorGap=1); group(method=”density”, bw=15, mzwid=0.015, minifrac=0.75, minsamp=3, max=50); fillPeaks(method=”chrom”).

Muma parameters: explore.data(scaling=”pareto”, scal=”TRUE”, normalize=TRUE, imputation=TRUE, imput=”half.minimum).

ST 1: Significantly altered features of GSD1 patient plasma compared to controls. Metabolites that were not in the internal library and were confirmed by fragment pattern matching are denoted with §.

| **Feature** | **Ion** | **Metabolite** | **Fold Change** | **Corr. p-value** |
| --- | --- | --- | --- | --- |
| 204.12292 | M+H | Acetylcarnitine | 1.47 | 7.98E-08 |
| 268.09561 | M+H | Adenosine | 0.30 | 9.73E-03 |
| 175.11881 | M+H | Arginine | 0.42 | 6.51E-33 |
| 213.06843 | M+K | Arginine | 0.28 | 1.10E-19 |
| 245.08444 | M+H | Biotine | 4.35 | 6.03E-19 |
| 162.10734 | M+H | Carnitine | 1.24 | 3.48E-04 |
| 104.10248 | M+ | Choline | 1.33 | 1.29E-07 |
| 159.06791 | M-NH3+H | Citrulline | 0.36 | 9.66E-25 |
| 176.10215 | M+H | Citrulline | 0.68 | 4.56E-03 |
| 170.02324 | M+K | Creatine | 3.39 | 5.64E-28 |
| 132.0748 | M+H | Creatine | 3.73 | 5.93E-24 |
| 154.04936 | M+Na | Creatine | 2.73 | 8.01E-05 |
| 133.06361 | M+13C+H | Creatine | 2.88 | 3.32E-03 |
| 114.06205 | M+H | Creatinine | 0.63 | 2.35E-17 |
| 244.08633 | M+H | Cytidine | 3.19 | 2.06E-19 |
| 115.04332 | M+H | Dihydrouracil | 0.73 | 4.41E-03 |
| 148.06057 | M+H | Glutamate | 4.67 | 3.34E-27 |
| 130.04616 | M-NH3+H | Glutamine | 0.64 | 6.65E-21 |
| 147.07446 | M+H | Glutamine | 0.72 | 1.75E-09 |
| 76.03368 | M+H | Glycine | 0.78 | 5.93E-03 |
| 118.05844 | M+H | Guanidinoacetate | 0.43 | 2.36E-21 |
| 136.04212 | M+H | Homocysteine | 3.94 | 7.87E-26 |
| 110.02226 | M+H | Hypotaurine | 0.63 | 3.13E-05 |
| 152.05105 | M+ACN+H | Hypotaurine | 0.67 | 1.25E-04 |
| 189.12762 | M+H | N-Methylarginine^§^ | 0.22 | 3.56E-20 |
| 169.08803 | M+Na | Lysine | 0.72 | 2.61E-03 |
| 150.05564 | M+H | Methionine | 0.63 | 1.22E-16 |
| 133.09458 | M+H | Ornithine | 0.50 | 3.87E-17 |
| 166.08386 | M+H | Phenylalanine | 0.71 | 5.17E-11 |
| 88.03414 | M-H2O+H | Serine | 0.59 | 1.10E-18 |
| 148.07345 | M+ACN+H | Serine | 0.33 | 3.87E-18 |
| 106.04542 | M+H | Serine | 0.62 | 6.03E-16 |
| 128.01882 | M+Na | Serine | 2.04 | 3.06E-04 |
| 249.0859 | M+H | Thymidine | 0.36 | 2.67E-05 |
| 127.04128 | M+H | Thymine | 0.81 | 2.40E-02 |
| 243.04483 | M+K | Tryptophan | 0.48 | 6.11E-14 |
| 139.04438 | M+H | Urocanate | 0.68 | 8.90E-03 |
| 118.0773 | M+H | Valine | 0.45 | 1.51E-17 |
| 145.01257 | M-H | a-Ketoglutarate | 4.44 | 8.23E-10 |
| 131.04455 | M-H | Asparagine | 0.60 | 1.30E-16 |
| 149.04384 | M-H | Pentose | 0.69 | 9.47E-16 |
| 87.00701 | M-H | pyruvate | 3.70 | 3.17E-34 |
| 117.01756 | M-H | Succinate | 0.66 | 5.99E-16 |
| 151.0209 | M-H | Xanthine | 7.52 | 5.47E-13 |
|  |  |  |  |  |

ST 2: Significantly altered features of GSD1a compared to GSD1b patients. Metabolites that were not in the internal library and were confirmed by fragment pattern matching are denoted with §.

| **Feature** | **Ion** | **Metabolite** | **Fold Change** | **Corr. p-value** |
| --- | --- | --- | --- | --- |
| 154.04936 | M+Na | Creatine | 0.34 | 1,65E-04 |
| 203.14006 | M+H | Dimethylarginine^§^ | 0.40 | 1,48E-10 |
| 147.07446 | M+H | Glutamine | 0.67 | 3,36E-06 |
| 130.04616 | M-NH3+H | Glutamine | 0.70 | 8,50E-04 |
| 244.15736 | M+H | Tiglylcarnitine^§^ | 2.00 | 2,33E-06 |
| 243.04483 | M+K | Tryptophan | 0.53 | 1,86E-02 |
| 157.03506 | M-H | Allantoin^§^ | 1.64 | 1,11E-02 |
|  |  |  |  |  |

ST 3: Significantly altered features of GSD1 patients with liver adenoma compared to patients without liver adenoma. Metabolites that were not in the internal library and were confirmed by fragment pattern matching are denoted with §.

| **Feature** | **Ion** | **Metabolite** | **Fold Change** | **Corr. p-value** |
| --- | --- | --- | --- | --- |
| 116.06651 | M+H | Proline | 1.53 | 1.30E-02 |
| 218.1279 | M+H | Propionylcarnitine^§^ | 2.84 | 4.60E-02 |
| 249.0859 | M+H | Thymidine | 0.25 | 3.55E-03 |
|  |  |  |  |  |

ST 4: Significantly altered features of GSD1 patients with microalbuminuria compared to patients without. Metabolites that were not in the internal library and were confirmed by fragment pattern matching are denoted with §.

| **Feature** | **Ion** | **Metabolite** | **Fold Change** | **Corr. p-value** |
| --- | --- | --- | --- | --- |
| 114.06205 | M+H | Creatinine | 1.48 | 7.62E-03 |
| 152.05105 | M+ACN+H | Hypotaurine | 1.78 | 3.17E-02 |
| 150.05564 | M+H | Methionine | 1.59 | 1.71E-04 |
| 166.08386 | M+H | Phenylalanine | 1.33 | 2.81E-02 |
| 218.1279 | M+H | Propionylcarnitine^§^ | 4.87 | 8.74E-06 |
| 243.04483 | M+K | Tryptophan | 2.05 | 1.55E-03 |
| 165.05372 | M-H2O+H | Tyrosine | 1.78 | 8.87E-06 |
| 136.07276 | M-CO-H2O+H | Tyrosine | 1.76 | 1.51E-05 |
| 204.06246 | M+H | Tyrosine | 4.07 | 1.01E-02 |
|  |  |  |  |  |

ST 5: Fatty acid profiles

|  | **GSD I patients** | **GSD Ia** | **GSD Ib** | **controls** |
| --- | --- | --- | --- | --- |
| Total (n) | 14 | 11 | 3 | 31 |
| Relative abundance^1)^ |  |  |  |  |
| 14:0 | 0.014 ± 0.005* | 0.016 ±0.004# | 0.009 ±0.006 | 0.001 ±0.004 |
| 16:0 | 0.289 ±0.020* | 0.290 ±0.023 | 0.287 ±0.008 | 0.249 ±0.025 |
| 16:1n7 | 0.049 ±0.017* | 0.051 ±0.018 | 0.039 ±0.003 | 0.009 ±0.010 |
| 18:0 | 0.064 ±0.007* | 0.064 ±0.007 | 0.063 ±0.010 | 0.081 ±0.011 |
| 18:1n9 | 0.351 ±0.037* | 0.345 ±0.037 | 0.375 ±0.033 | 0.264 ±0.059 |
| 18:1n7 | 0.019 ±0.004 | 0.019 ±0.004 | 0.016 ±0.001 | 0.019 ±0.059 |
| 18:2n6 | 0.168 ±0.034* | 0.163 ±0.036 | 0.183 ±0.027 | 0.340 ±0.056 |
| 20:3n6 | 0.008 ±0.003* | 0.008 ±0.003 | 0.005 ±0.003 | 0.001 ±0.002 |
| 20:4n6 | 0.034 ±0.012 | 0.037 ±0.011 | 0.023 ±0.010 | 0.037 ±0.014 |
| 22:6n3 | 0.002 ±0.003* | 0.003 ±0.003 | 0. 00 | 0.00 |
| Molar ratios^2)^ |  |  |  |  |
| Ratio 16:0/18:2n6 | 2.49 ±0.65* | 2.58 ±0.68 | 2.18 ±0.45 | 0.94 ±0.27 |
| Ratio 16:1/16:0 | 0.149 ±0.044* | 0.156 ±0.048 | 0.123 ±0.009 | 0.032 ±0.036 |
| Ratio 18:1n9/18:0 | 5.07 ±0.99* | 4.96 ±0.97 | 5.48 ±1.19 | 2.93 ±0.76 |

Fatty acid species measured clearly above detection limit are shown

^1)^ Relative abundance expresses the relative contribution of an individual fatty acid to the total fatty acid pool, while

^2)^ molar ratios express the ratio of two individual fatty acids, calculated from the molar concentrations of the respective fatty acids

*significant differences for comparison of GSD1 versus controls p < 0.001

# significant difference for comparison GSD1a vs GSD1b p < 0.05


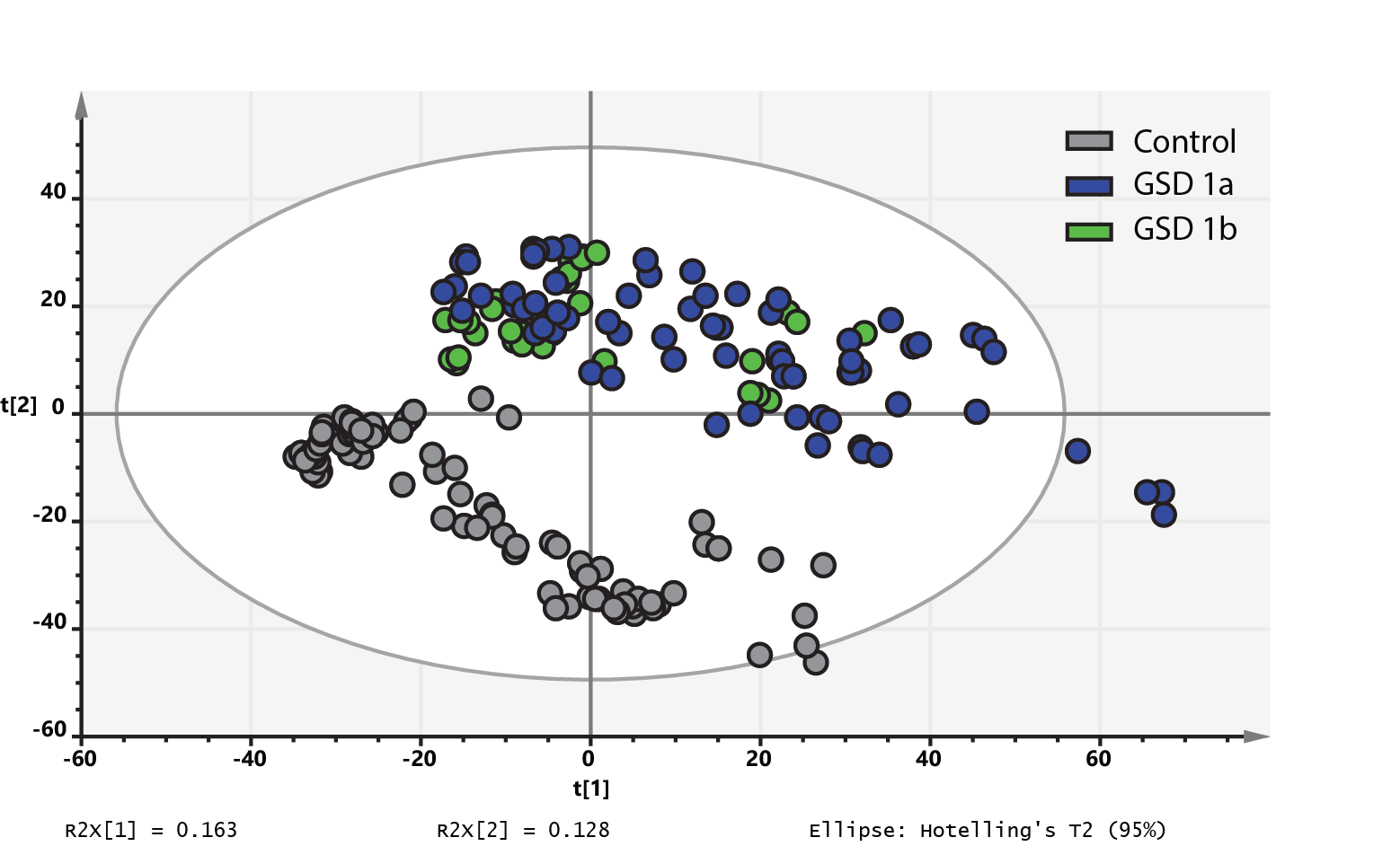


**Figure S1:** Unsupervised principal component analysis (PCA) of GSD Ia (blue) and GSD Ib (green) patient plasma samples versus healthy controls (grey points) in negative mode, with R2X[1] displaying the interpretable degree of the first principle component (horizontal) and R2X[2] displaying the interpretable degree of the second principle component (vertical). All samples are shown as technical triplicates.
